# Supplementary material for: PheWAS-based clustering of Mendelian Randomisation instruments reveals distinct mechanism-specific causal effects between obesity and educational attainment
Source: Nat Commun. 2024 Feb 15;15:1420. doi: 10.1038/s41467-024-45655-8 (PMC10869347; doi:10.1038/s41467-024-45655-8)
Supplement: Supplementary file 1 — Supplementary Information [file 41467_2024_45655_MOESM1_ESM.pdf]

Supplementary Information

Supplementary Figures

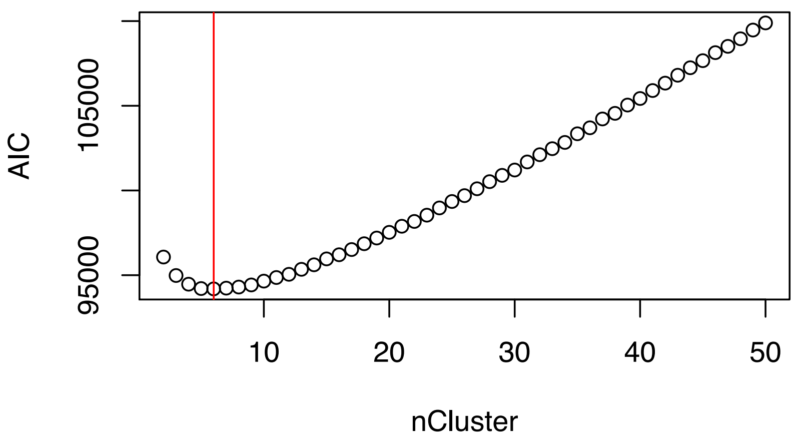

Supplementary Figure 1: **Dot plot representing the corresponding Akaike Information Criterion scores across varying K-means centres for BMI.** K-means centres vary from 2 to 50 clusters. The red vertical line represents the number of centres/cluster with the lowest score.

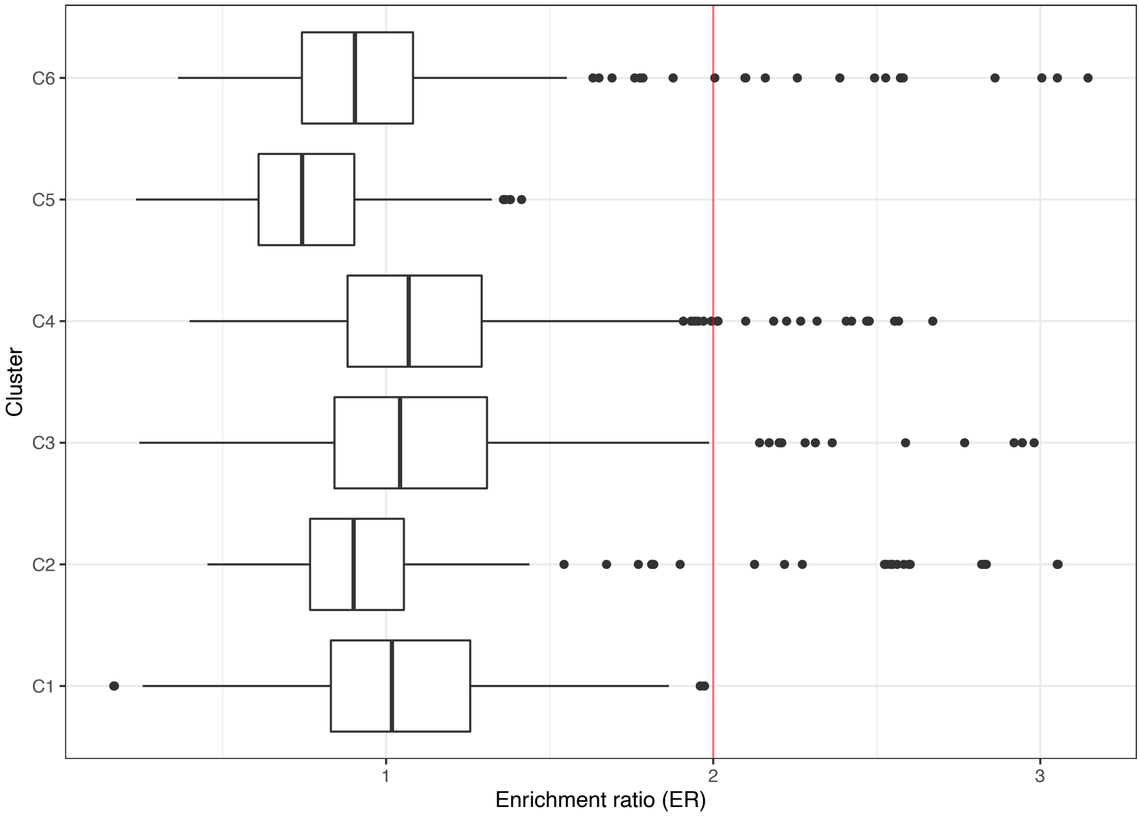

Supplementary Figure 2: **Boxplot showing the enrichment ratio of all traits in each cluster.** BMI IVs have been clustered into 6 clusters using K-means. The enrichment ratio of each trait calculated using the cluster-specific IVs is shown in the boxplot. In the boxplots, the lower and upper hinges correspond to the first and third quartiles, the middle bar corresponds to the median, whereas the upper whisker is the largest data point smaller than 1.5× inter-quartile range above the third quartile. The lower whisker is defined analogously.

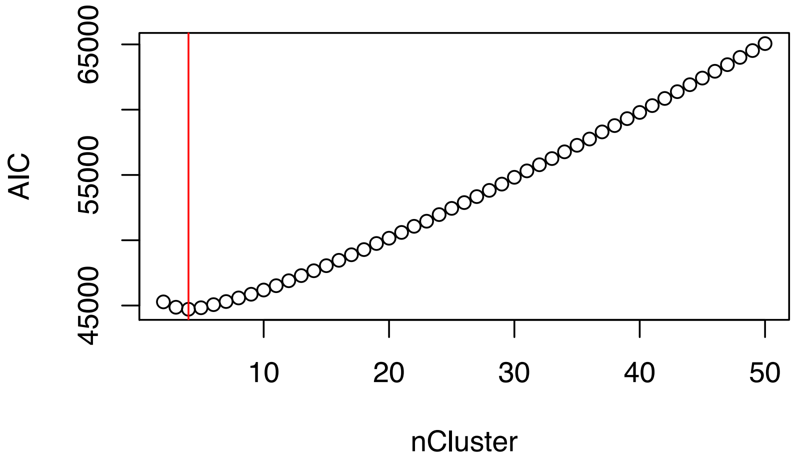

Supplementary Figure 3: **Dot plot representing the corresponding Akaike Information Criterion scores across varying K-means centres for child BMI.** K-means centres vary from 2 to 50 clusters. The red vertical line represents the number of centres/cluster with the lowest score.

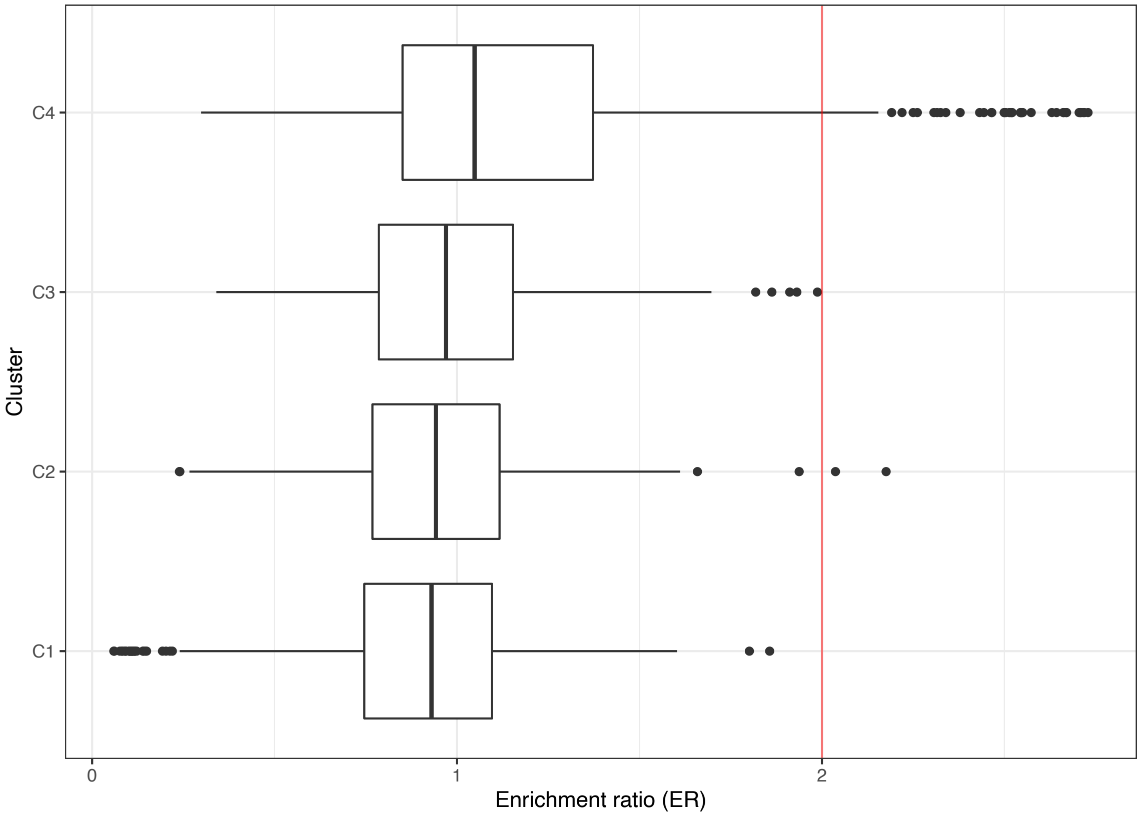

Supplementary Figure 4: **Boxplot showing the enrichment ratio of all traits in each cluster.** Child BMI IVs have been clustered into 4 clusters using K-means. The enrichment ratio of each trait calculated using the cluster-specific IVs is shown in the barplot. In the boxplots, the lower and upper hinges correspond to the first and third quartiles, the middle bar corresponds to the median, whereas the upper whisker is the largest data point smaller than 1.5× inter-quartile range above the third quartile. The lower whisker is defined analogously.

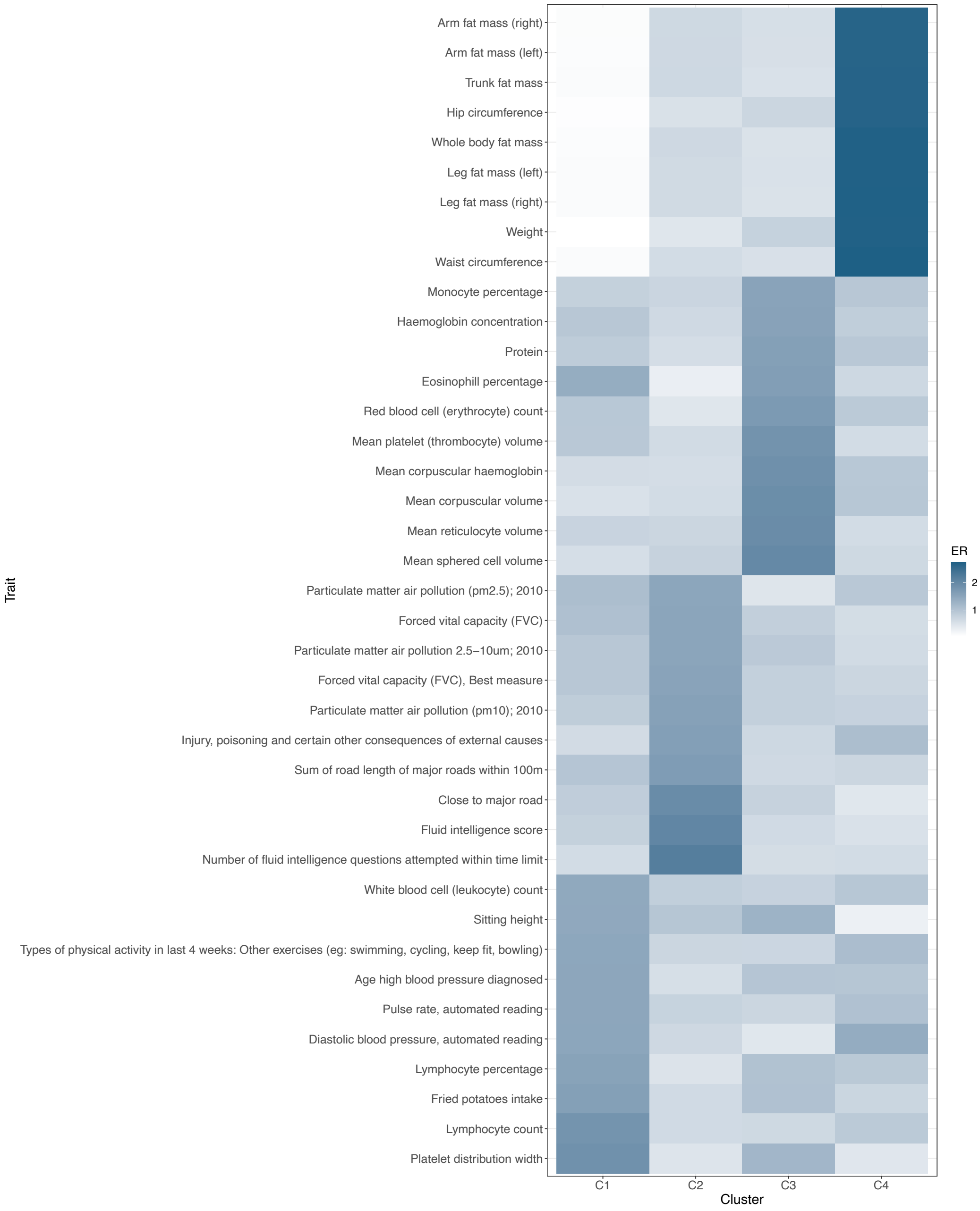

Supplementary Figure 5: **Heatmap of the enrichment ratio of the top 10 traits in each cluster.** Body size at age 10 is used as a proxy exposure trait for child BMI. K-means clustering revealed 4 clusters with the following trait enrichment ratios.

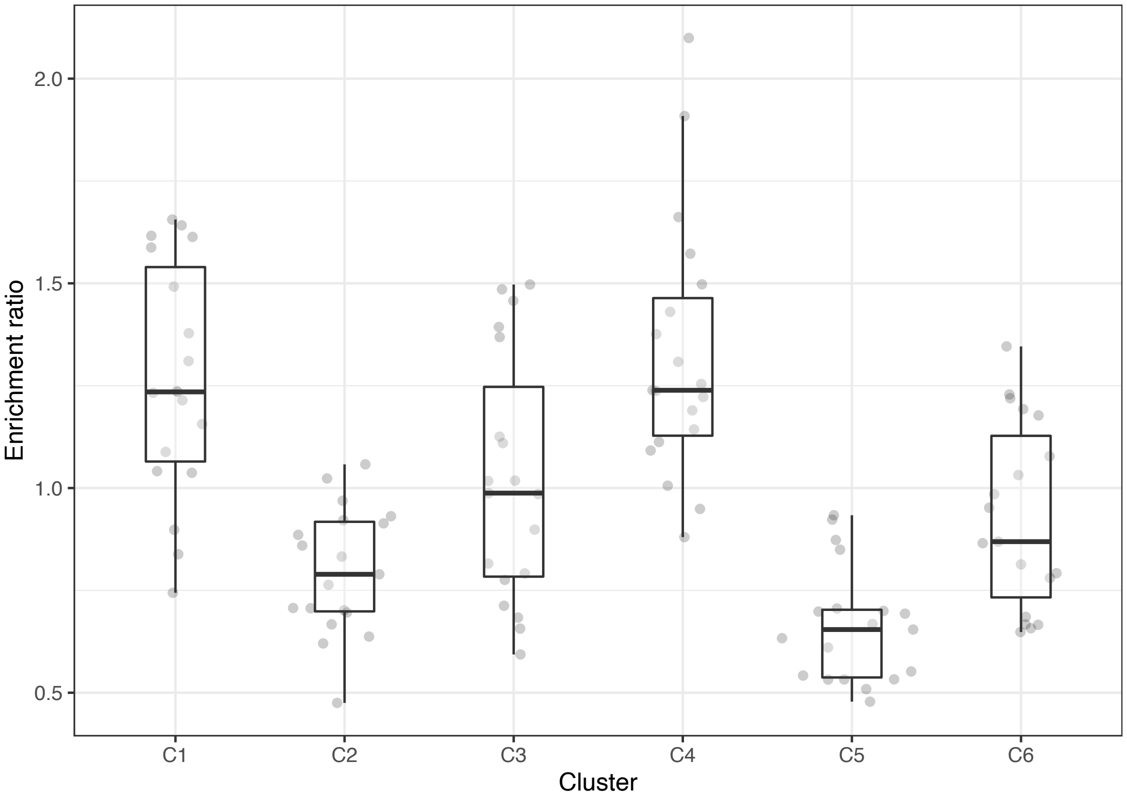

Supplementary Figure 6: **Boxplot showing the ER for confounder traits across the clusters.** Confounder traits were categorised in a systematic search. In the boxplots, the lower and upper hinges correspond to the first and third quartiles, the middle bar corresponds to the median, whereas the upper whisker is the largest data point smaller than 1.5× inter-quartile range above the third quartile. The lower whisker is defined analogously.

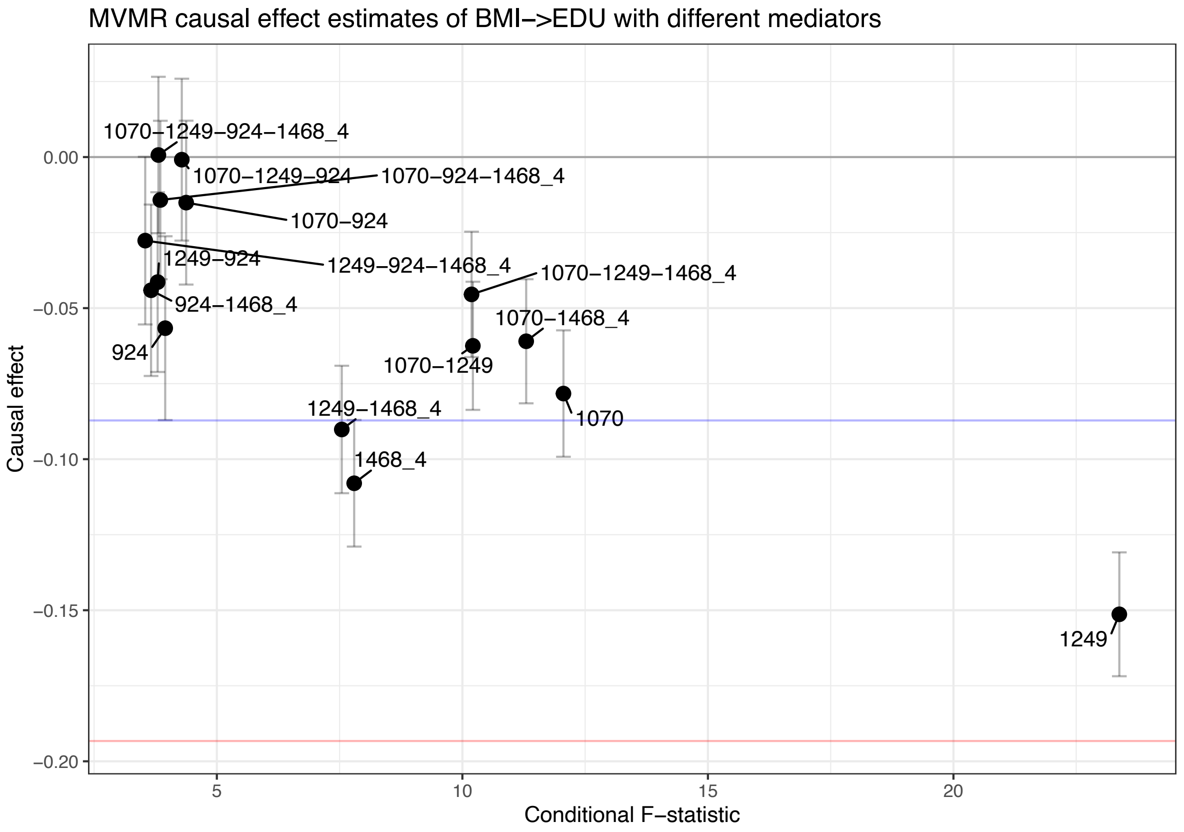

Supplementary Figure 7: **Dot plot showing the causal effect estimate of BMI on EDU conditional on various combinations of three candidate confounder traits.** Vertical error bars represent the point estimate $\pm 1.96\times$ standard error (SE). The blue horizontal line represents the observational correlation between BMI and EDU, whereas the red horizontal line represents the univariate causal effect estimate of BMI on EDU. Trait 1070: 'Time spent watching television (TV)', trait 924: 'Usual walking pace', trait 1249: 'Past tobacco smoking', 1468_4: 'Cereal type: Muesli'.

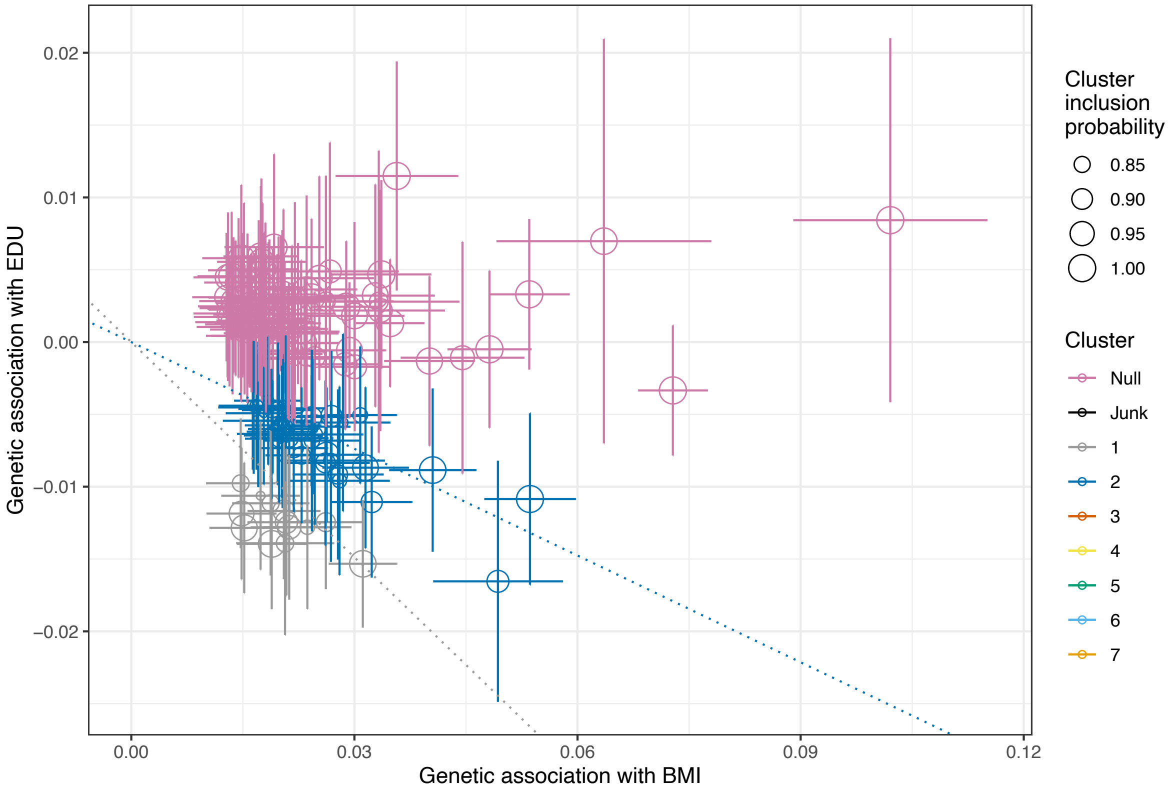

Supplementary Figure 8: **Dot plot showing the genetic association of IVs with the exposure: BMI, and the outcome: EDU. The exposure IVs have been clustered using MR-Clust based on their similarity in causal effect estimates.** MR-Clust has revealed 2 main clusters for BMI's causal effect on EDU as well as a 'null' cluster. The IVs plotted have a cluster inclusion probability greater than or equal to 80%. The slopes represent the causal effect estimate of each cluster.

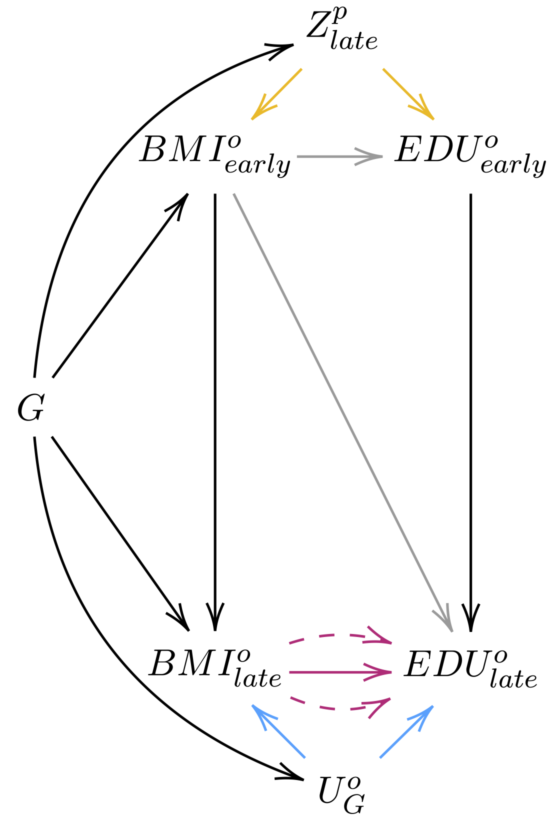

Supplementary Figure 9: **Directed Acyclic Graph (DAG) illustrating the relationship between BMI and EDU.** The DAG involves early and later-in-life (late) versions of BMI as the exposure trait and EDU as the outcome trait. G represents genetic instruments, U^o_G represents a heritable confounder acting on the trait pair, whereas Z represents a parental trait involved in exerting dynastic effects. The superscripts p and o stand for parental and offspring respectively, and the dashed arrows from X to Y represent the different biological mechanisms through which a causal effect can emerge.Grey arrows represent possible causal pathways between the early traits as well as early BMI and late EDU.

Supplementary Methods

1.1 Different model selection criteria and additional number of clusters

In order to test for multiple model selection criteria, we tested for the optimal cluster number using both AIC (as shown in the manuscript) and Bayesian information criterion (BIC).
Using BIC, we end up with 2 clusters being the optimal for BMI SNPs with heterogeneous causal effects on EDU (cluster #1 = -0.13 (p-value = 6.61E-16), cluster #2 = -0.34 (p-value = 6.15E-44)), and their enrichment reflects a clear distinction between enrichment for lean-mass and body related traits in cluster #1 and a mixed bag of trait enrichment for cluster #2 including lung/height/blood and SES-proxy traits.

This result is due to BIC introducing a stronger penalty term, $k \times \log(n)$, where k is the number of clusters and n corresponds to the number of (independent) samples used.
However, in our case n represents the number of traits, which are highly correlated. Also, the more traits are used to cluster the SNPs, the more clusters we expect to obtain as they allow for a more fine-grain resolution of the underlying biological mechanisms. For these reasons, we do not believe that BIC is an appropriate measure to quantify clustering fit in this situation. Therefore, the BIC-based selection of optimal cluster number does not alter the main message/result, and only leads to coarser grain clusters.

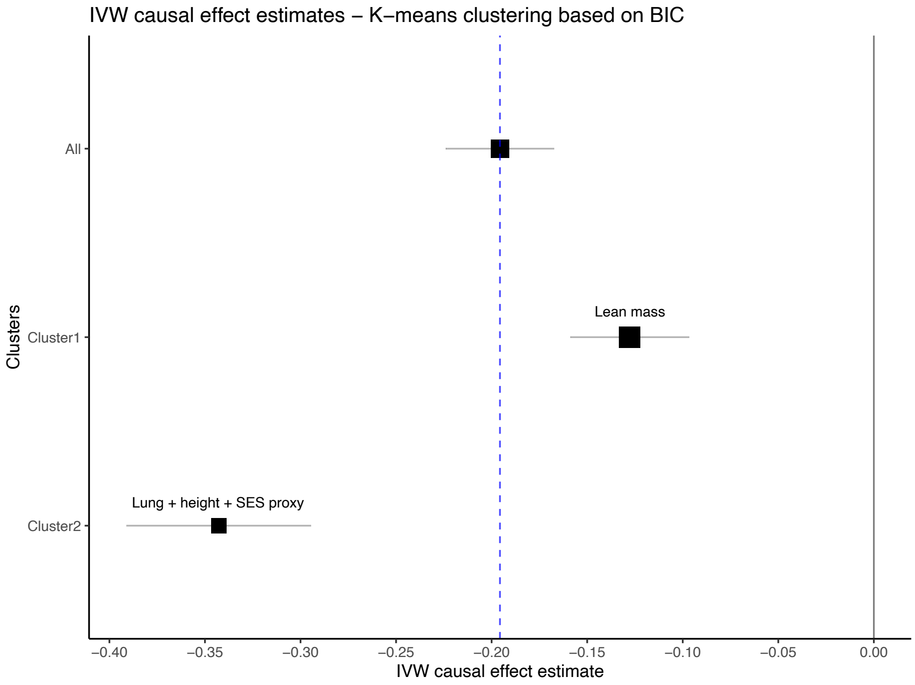

Supplementary Figure 10: **Causal effect estimates of BIC-clustered BMI SNPs on educational attainment.** Cluster #1 is enriched for traits related to lean mass, whereas cluster #2 is enriched for a combination of lung, height and SES-proxy traits. Horizontal error bars represent the point estimate $\pm 1.96\times$ standard error (SE). The blue vertical line represents the causal effect estimated using all BMI IVs. Box sizes of clusters represent the proportion of the number of IVs in each cluster to the total.

On the other hand, we also tried to forcibly increase the number of clusters to 8 in the hopes of achieving more distinction in enrichment. We observed similar heterogeneous causal effects on EDU, where the smallest and largest effects were from clusters enriched for lean mass and SES-related traits respectively. As for the rest of the clusters, another 2 were strongly enriched for food supplements and a mix of height/blood/lung measurement traits, another was enriched for a mix of diseases and three other clusters had low enrichments for miscellaneous traits.

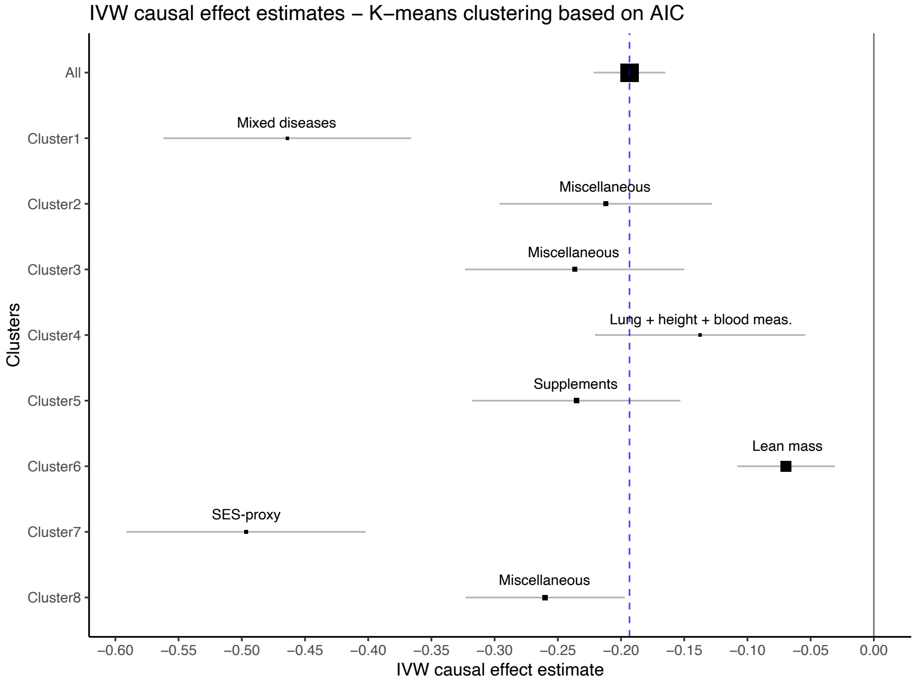

Supplementary Figure 11: **Causal effect estimates of clustered (forced 8 clusters) BMI SNPs on educational attainment.** The trait-enrichment of each cluster is labelled. Horizontal error bars represent the point estimate $\pm 1.96\times$ standard error (SE). The blue vertical line represents the causal effect estimated using all BMI IVs. Box sizes of clusters represent the proportion of the number of IVs in each cluster to the total.

1.2 Using a coarsened variable as an exposure for MR: Comparative body size at age 10

To validate our comparison between the magnitude of effect estimates for adult and childhood BMI, given that childhood BMI was proxied by coarsened variable (Comparative body size at age 10), we ran the following analysis:

We simulated polygenic risk score (PRS) to explain 10% of childhood BMI and added Gaussian noise to generate childhood BMI values for 350,000 individuals. Individuals were then split into three categories, matching the proportion of plumper and skinnier subjects in the UK Biobank data. We then normalised this coarsened/trichotomized phenotype to have a variance of 1 (mimicking our original analysis). Both the real and the coarsened childhood BMI were regressed onto the PRS. Next, we simulated a continuous EDU score with true childhood BMI having a small (-0.1) causal effect on it. Finally, we ran MR for both the coarsened and the true childhood BMI on EDU, and compared the magnitudes of the causal effects of 100 different runs (figure below).

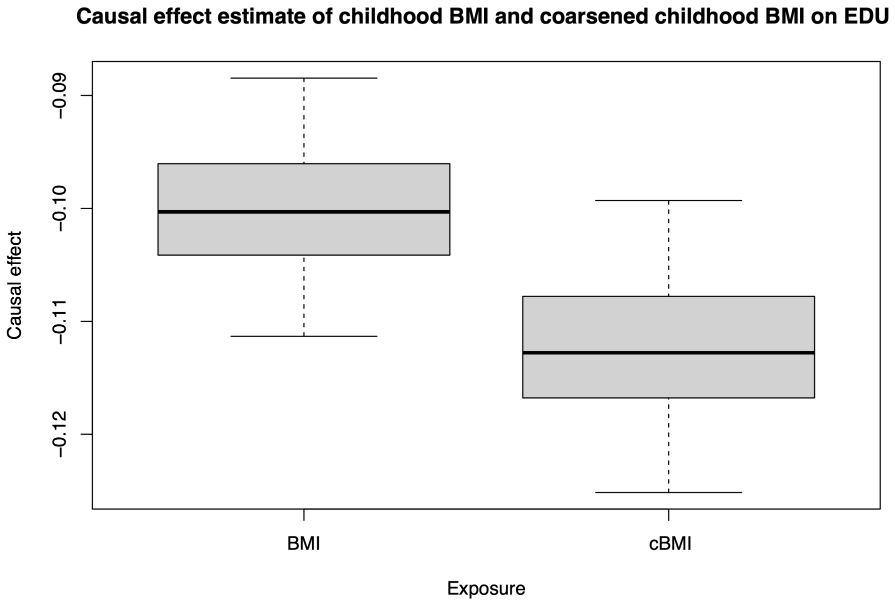

Supplementary Figure 12: **Causal effect estimate of childhood BMI and coarsened childhood BMI on EDU. True causal effect of childhood BMI on EDU is -0.1.** In the boxplots, the lower and upper hinges correspond to the first and third quartiles, the middle bar corresponds to the median, whereas the upper whisker is the largest data point smaller than 1.5× inter-quartile range above the third quartile. The lower whisker is defined analogously.

As seen in the results above, the causal effect estimates of BMI and coarsened BMI (cBMI) on EDU are comparable, with a slight (10%) increase of the average causal effect of cBMI in comparison to BMI's effect. This indicates that using a coarsened version of childhood BMI may have led to a slight overestimation of the causal effect, therefore the true childhood BMI on EDU effect may be even smaller than the estimated one (-0.03, 95% CI: $-0.06, 0$). Furthermore, we see that 1 SD change in cBMI is equivalent to 0.9 SD change in BMI, assuring us of the robustness of our results and data used.

1.3 Past tobacco smoking as a candidate confounder of the BMI-EDU relationship

Despite it being a candidate confounder trait, past tobacco smoking is unlikely to have a retroactive effect on education (or an effect at all, unlike education's effect on smoking). To further investigate this, we added the trait Smoking Initiation (GWAS obtained from Saunders et al.[1]), which on average occurs around the age of 17 in the UK population, to the MVMR analysis. We repeated first the stepwise-MVMR, obtained 'Smoking initiation', 'Time spent watching television (TV)', 'Cereal type: Muesli', and 'Usual walking pace' as candidate confounder traits with significant causal effects on EDU. Note that smoking initiation replaced past tobacco smoking in this step, as it no longer had a strong causal effect on EDU. Adding BMI to this set of exposures and then calculating its conditional F-statistic with their various combination, we discover that the combination of the first three traits give a conditional F-statistic ≥ 10 (12.53) and that BMI's conditional causal effect is severely attenuated, as shown in the table below:

Smoking initiation, as seen, has a significantly negative causal effect on education, but we would like to iterate that it, as well as the other candidate confounder traits are not necessarily true confounders, but are very likely to be proxies for a confounding parental environment/trait.

Phenotype	Description	α estimate	SE	P
SmkInit	Smoking initiation	-0.1358	0.0122	7.66E-27
1070	Time spent watching television (TV)	-0.2617	0.0238	2.91E-26
1468_4	Cereal type: Muesli	0.2920	0.0341	5.39E-17
21001_irnt	Body mass index (BMI)	-0.0383	0.0103	2.01E-04

Supplementary Table 1: MVMR analysis results of BMI and three candidate confounder traits on education. α: causal effect estimate.

Supplementary References

1. Saunders, G. R. B. *et al.* Genetic diversity fuels gene discovery for tobacco and alcohol use. *Nature* **612,** 720–724 (2022).
