# Supplementary material for: PheWAS-based clustering of Mendelian Randomisation instruments reveals distinct mechanism-specific causal effects between obesity and educational attainment
Source: Nat Commun. 2024 Feb 15;15:1420. doi: 10.1038/s41467-024-45655-8 (PMC10869347; doi:10.1038/s41467-024-45655-8)
Supplement: Supplementary file 3 — Description of Additional Supplementary Files [file 41467_2024_45655_MOESM3_ESM.pdf]

Description of Additional Supplementary Files

File Name: Supplementary Data 1
Description: Overview of data used in clustering, mendelian randomisation (MR) and colocalization analyses

File Name: Supplementary Data 2
Description: BMI IV RSIDs clustered into 6 clusters based on the lower score obtained from AIC

File Name: Supplementary Data 3
Description: Top 10 enrichment ratio traits for each cluster (BMI)

File Name: Supplementary Data 4
Description: TwoSampleMR results of BMI's causal effect on EDU using all IVs, as well as IVs from each cluster

File Name: Supplementary Data 5
Description: childBMI IV RSIDs clustered into 6 clusters based on the lower score obtained from AIC

File Name: Supplementary Data 6
Description: Top 10 enrichment ratio traits for each cluster (childBMI)

File Name: Supplementary Data 7
Description: TwoSampleMR results of childBMI's causal effect on EDU using all IVs, as well as IVs from each cluster

File Name: Supplementary Data 8
Description: TwoSampleMR results of BMI's causal effect on SBP using all IVs, as well as IVs from each cluster

File Name: Supplementary Data 9
Description: Candidate confounder traits with significantly larger TwoSampleMR causal effects on exposure (onEXP) and on outcome (onOUT), using one-sided t-test (p-value threshold 0.05)

File Name: Supplementary Data 10
Description: Candidate confounder traits with significantly larger TwoSampleMR causal effects on exposure (onEXP) and on outcome (onOUT), using one-sided t-test (stringent p-value threshold 0.01)

File Name: Supplementary Data 11
Description: Candidate confounder traits with significantly larger TwoSampleMR causal effects on exposure (onEXP) and on outcome (onOUT), using one-sided t-test (lenient p-value threshold 0.1)

File Name: Supplementary Data 12
Description: Variants with significant evidence for colocalisation with adipose tissue (PP.H4>=0.8)

File Name: Supplementary Data 13
Description: Variants with significant evidence for colocalisation with brain tissue (PP.H4>=0.8)
