# Supplementary material for: PheWAS-based clustering of Mendelian Randomisation instruments reveals distinct mechanism-specific causal effects between obesity and educational attainment
Source: Nat Commun. 2024 Feb 15;15:1420. doi: 10.1038/s41467-024-45655-8 (PMC10869347; doi:10.1038/s41467-024-45655-8)
Supplement: Supplementary file 5 — Reporting Summary [file 41467_2024_45655_MOESM5_ESM.pdf]

nature portfolio

Corresponding author(s): Liza Darrous

Last updated by author(s): Jan 8, 2024

Reporting Summary

Nature Portfolio wishes to improve the reproducibility of the work that we publish. This form provides structure for consistency and transparency in reporting. For further information on Nature Portfolio policies, see our Editorial Policies and the Editorial Policy Checklist.

Statistics

For all statistical analyses, confirm that the following items are present in the figure legend, table legend, main text, or Methods section.

n/a	Confirmed	
☐	☒	The exact sample size (*n*) for each experimental group/condition, given as a discrete number and unit of measurement
☐	☒	A statement on whether measurements were taken from distinct samples or whether the same sample was measured repeatedly
☐	☒	The statistical test(s) used AND whether they are one- or two-sided<br>*Only common tests should be described solely by name; describe more complex techniques in the Methods section.*
☐	☒	A description of all covariates tested
☐	☒	A description of any assumptions or corrections, such as tests of normality and adjustment for multiple comparisons
☐	☒	A full description of the statistical parameters including central tendency (e.g. means) or other basic estimates (e.g. regression coefficient) AND variation (e.g. standard deviation) or associated estimates of uncertainty (e.g. confidence intervals)
☐	☒	For null hypothesis testing, the test statistic (e.g. *F*, *t*, *r*) with confidence intervals, effect sizes, degrees of freedom and *P* value noted<br>*Give P values as exact values whenever suitable.*
☒	☐	For Bayesian analysis, information on the choice of priors and Markov chain Monte Carlo settings
☒	☐	For hierarchical and complex designs, identification of the appropriate level for tests and full reporting of outcomes
☐	☒	Estimates of effect sizes (e.g. Cohen's *d*, Pearson's *r*), indicating how they were calculated

Our web collection on statistics for biologists contains articles on many of the points above.

Software and code

Policy information about availability of computer code

Data collection	No software was used for data collection.
Data analysis	Data analysis was performed using code written in the freely available R programming language (R versions 4.2.2 and 4.1.3). All the scripts as well as a working-example can be found on https://github.com/LizaDarrous/PheWAS-cluster. We also used the following R-package for data analysis: 'TwoSampleMR' (version 0.5.5, freely available from https://github.com/MRCIEU/TwoSampleMR), and 'coloc' (version 5.2.3, freely available from https://cran.r-project.org/web/packages/coloc/index.html).

For manuscripts utilizing custom algorithms or software that are central to the research but not yet described in published literature, software must be made available to editors and reviewers. We strongly encourage code deposition in a community repository (e.g. GitHub). See the Nature Portfolio guidelines for submitting code & software for further information.

Data

Policy information about availability of data

All manuscripts must include a data availability statement. This statement should provide the following information, where applicable:

- Accession codes, unique identifiers, or web links for publicly available datasets
- A description of any restrictions on data availability
- For clinical datasets or third party data, please ensure that the statement adheres to our policy

The origin and unique identifier of each of the summary statistics data used is referenced in Supplementary Data 1.

The UK Biobank summary statistics data used in this study can be downloaded from http://www.nealelab.is/uk-biobank. BMI meta-analyzed GWAS and adipose meta-analyzed cis-eQTL can be obtained from Leyden et. al 2022, and the brain cis-eQTL data can be downloaded from https://yanglab.westlake.edu.cn/software/smr/#DataResource .

Research involving human participants, their data, or biological material

Policy information about studies with human participants or human data. See also policy information about sex, gender (identity/presentation), and sexual orientation and race, ethnicity and racism.

Reporting on sex and gender	There was no separate analysis done based on either sex or gender in our study. Our analysis focused mainly on the genetics of a certain trait and the various underlying (causal) pathways it could have on another trait/outcome.
Reporting on race, ethnicity, or other socially relevant groupings	Summary statistics from Neale lab were obtained from GWAS analysis ran in White British individuals from the UK Biobank.
Population characteristics	Summary statistics from Neale lab were obtained from GWAS analysis ran in White British individuals from the UK Biobank. The participants of the UK Biobank were aged between 40-69, relatively healthy.
Recruitment	Participants in the UK Biobank were voluntarily recruited at various health centers, for more details please see: https://www.ukbiobank.ac.uk/enable-your-research
Ethics oversight	Please see: https://www.ukbiobank.ac.uk/learn-more-about-uk-biobank/about-us/ethics

Note that full information on the approval of the study protocol must also be provided in the manuscript.

Field-specific reporting

Please select the one below that is the best fit for your research. If you are not sure, read the appropriate sections before making your selection.

☒ Life sciences ☐ Behavioural & social sciences ☐ Ecological, evolutionary & environmental sciences

For a reference copy of the document with all sections, see nature.com/documents/nr-reporting-summary-flat.pdf

Life sciences study design

All studies must disclose on these points even when the disclosure is negative.

Sample size	Sample sizes were reported in GWAS summary statistics and used as needed in our analysis. Effective sample size for case-control GWAS summary statistics was calculated using N_eff = (4*Ncase*Ncontrol)/(Ncase + Ncontrol).
Data exclusions	Analysis for traits with effective sample size smaller than 50'000 was dropped, similarly some SNPs were excluded based on MR assumption violations. SNP data of those falling in the HLA region of chromosome 6 were excluded due to the abundance of SNPs associated with autoimmune and infectious diseases as well as the complicated LD structure present in that region. This exclusion criteria was pre-established, and is commonly done in GWAS analysis studies.
Replication	The results are fully reproducible with the code available.
Randomization	There was no group assignment performed in this study, since it was not experimental.
Blinding	Blinding is not relevant to our study since it was not experimental, and all data collection and processing was done in previous studies.

Reporting for specific materials, systems and methods

We require information from authors about some types of materials, experimental systems and methods used in many studies. Here, indicate whether each material, system or method listed is relevant to your study. If you are not sure if a list item applies to your research, read the appropriate section before selecting a response.

Materials & experimental systems

n/a	Involved in the study
☒	☐ Antibodies
☒	☐ Eukaryotic cell lines
☒	☐ Palaeontology and archaeology
☒	☐ Animals and other organisms
☒	☐ Clinical data
☒	☐ Dual use research of concern
☒	☐ Plants

Methods

n/a	Involved in the study
☒	☐ ChIP-seq
☒	☐ Flow cytometry
☒	☐ MRI-based neuroimaging
